# Patient Satisfaction in Rural Versus Non‐Rural US Hospitals

**DOI:** 10.1002/puh2.219

**Published:** 2024-07-17

**Authors:** Sharon Vu, Logan Reese, Neha Patel, Man Hung

**Affiliations:** ^1^ Boston University Graduate Medical Science Boston Massachusetts USA; ^2^ Roseman University of Health Sciences College of Dental Medicine South Jordan Utah USA; ^3^ Department of Orthopaedic Surgery Operations University of Utah Salt Lake City Utah USA; ^4^ School of Business University of Utah Salt Lake City Utah USA; ^5^ Department of Veterans Affairs Medical Center Salt Lake City Utah USA

**Keywords:** HCAHPS, healthcare quality, patient satisfaction, regional inequality, rural status

## Abstract

**Introduction:**

Assessing patient satisfaction is an effective way to provide an evaluation of the quality of healthcare services. As a result, the Hospital Consumer Assessment of Healthcare Providers and Systems (HCAHPS) Patient Survey was established as the first standardized national survey to publicly report patient ratings on hospital care. There remains a lack of research that examines rural–urban distinctions in patient satisfaction. The objective of this study was to investigate if there is a difference in hospital determinants of patient satisfaction among hospitals based on rural status.

**Methods:**

The study utilized two public datasets containing HCAHPS survey results from 4714 US hospitals and from eligible cancer hospitals that participate in the Prospective Payment System‐Exempt Cancer Hospital Quality Reporting. The data were collected from July 1, 2021 to June 30, 2022 and published by Centers for Medicare & Medicaid Services. Rural status of each hospital was determined by matching zip codes with data provided by the Health Resources & Services Administration.

**Results:**

A total of 3248 hospitals were included in the analysis, with approximately 51.14% located in rural areas. There was a significant difference across every hospital determinant when comparing rural and non‐rural status. Rural hospitals had a higher score for every hospital determinant (Cleanliness, Nurse communication, Doctor communication, Staff responsiveness, Communication about medicines, Discharge information, Care transition, Overall hospital rating, Quietness, and Willingness to recommend the hospital), indicating better performance.

**Conclusion:**

Rural hospitals demonstrated better HCAHPS performance across every hospital determinant in comparison to urban hospitals, suggesting possible recent improvements. Future research is necessary to investigate this trend and its consistency, which can inform targeted interventions to better address the healthcare needs of diverse populations in the United States.

## Graphical abstract

Rural hospitals demonstrated better HCAHPS performance across every hospital determinant in comparison to urban hospitals. Future research is necessary to investigate this trend and its consistency, which can inform targeted interventions to better address the healthcare needs of diverse populations in the United State.

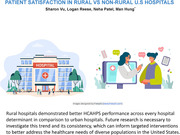

## 1 Introduction

There is a consensus and strong emphasis on utilizing patient satisfaction as a quality indicator because it is a realistic, cost‐effective, and time‐efficient approach [1, 2]. Assessing patient satisfaction helps identify areas for improvement in healthcare services, such as resource utilization, communication between healthcare providers and patients, and safety enhancements [3]. For example, prolonged wait times and delays were found to be the strongest predictor for dissatisfaction among emergency department patients and are an area that should be targeted for remediation [4]. Simultaneously, it is crucial to align healthcare services with patients’ expectations to enhance their overall experience, as studies have shown that satisfied patients are more likely to follow their physician's advice and exhibit stronger loyalty to their healthcare providers [5, 6].

Patient satisfaction can be measured by using quantitative methods, which usually involve questionnaire surveys. These are more commonly used as they allow for large sample sizes and can facilitate statistical testing for association and comparability. To successfully understand and improve patients’ experience, what is measured must reflect what patients care about most [3]. In this study, the Hospital Consumer Assessment of Healthcare Providers and Systems (HCAHPS) Patient Survey was utilized, as most US hospitals participated to receive their Medicare reimbursement. The HCAHPS is guided by three key objectives through public reporting: enabling objective hospital comparisons on patient‐relevant topics, incentivizing hospitals to improve quality, and enhancing healthcare accountability by increasing transparency in the provided healthcare quality in exchange for public funding [7].

As patient satisfaction continues to evolve, ongoing monitoring of the determinants related to current patient satisfaction is essential [8]. Researchers have also emphasized the importance of comparing studies that employ consistent survey questions and response options with exact wording to ensure comparability across studies [9, 10]. It is challenging to make broad and definitive conclusions when studies use different survey methods to measure patient satisfaction. Given that HCAHPS surveys are standardized, widely used by hospitals in the United States, and have annual updates to the results, reviewing publicly available HCAHPS data enables both the identification of areas for quality improvement and the comparison of performance across different hospitals nationally.

Furthermore, it is essential to examine geographical disparities in patient satisfaction, considering where patients access care. Conducting geographical comparisons through tools like HCAHPS surveys provides insight into the geographic factors, such as rural status, that contribute to variations in healthcare quality across different regions of the United States. For example, one study that compared patient satisfaction between rural and non‐rural hospitals found that rural hospitals demonstrated lower levels of cleanliness but higher levels of staff responsiveness and quietness in their facilities [11]. However, it only analyzed three patient satisfaction measures of the HCAHPS surveys (responsiveness of staff, cleanliness, and quietness of hospital) and was limited to the state of Massachusetts [11]. A 2012 study revealed that hospitals with lower performance on the HCAHPS surveys tended to have fewer beds, a lower nurse‐to‐patient ratio, and were more likely to be southern‐region safety‐net facilities in rural areas, in contrast to hospitals performing at intermediate and high levels [12]. However, another study using HCAHPS surveys found that among orthopedic patients at an academic medical center in the United States, those who lived in less affluent zip codes were more willing to recommend the hospital and give a higher overall hospital rating compared to patients who lived in wealthier zip codes [13].

This emphasis on geographical disparities is particularly important because multiple studies have highlighted that residents of rural areas experience disproportionately worse health outcomes than their urban counterparts in the United States [14, 15, 16]. Residents of rural areas tend to have higher rates of morbidity, mortality, smoking, obesity, and opiate‐use disorder than those of urban areas [17]. These disparities are likely related to the financial and workforce resource limitations while being paid based on a fee‐for‐service model [18]. Consequently, it is essential to have rural representation in policy‐making, as many current policies are based on the urban majority.

Despite this emphasis on addressing geographical disparities, there is a notable lack of research that examines rural–urban distinctions and their impact on hospital determinants of patient satisfaction. The use of HCAHPS surveys can provide insights into how patient satisfaction is influenced by these geographical disparities, helping policymakers and healthcare providers better tailor their services to meet the unique needs of different populations in the United States. Therefore, the goal of this study is to investigate whether rural status influences patient satisfaction ratings.

## 2 Methods

### 2.1 Settings

Participation in the HCAHPS varies based on the hospital type and is open to all patients. Under the Inpatient Prospective Payment System (IPPS), acute care hospitals are required to collect and submit HCAHPS data for a full annual payment update, whereas acute care hospitals that are not under the IPPS may participate voluntarily. The IPPS is a payment system established under Section 1886(d) of the Social Security Act for Medicare Part A [19]. Under IPPS, hospitals are initially paid by Centers for Medicare & Medicaid Services (CMS) based on a standardized payment rate for each case or diagnosis rather than for the actual costs incurred during a patient's stay [19]. The remaining Medicare reimbursement is adjusted based on domains like clinical processes, patient outcomes, and patient experience. Two percent of the overall Medicare reimbursements is based on the HCAHPS survey results [20, 21]. Thus, hospitals with lower HCAHPS performance will receive lower reimbursement from CMS [20]. The Deficit Reduction Act of 2005 incentivized acute care hospitals to participate in HCAHPS, and the Patient Protection and Affordable Care Act of 2010 strengthened the incentive for IPPS hospitals to improve patient experience [7].

The survey comprises 29 questions that ask discharged patients about their recent hospital stay [7]. Of these questions, 19 specifically address crucial aspects of the hospital experience, such as communication with health providers, responsiveness of hospital staff, cleanliness and quietness of the hospital environment, and overall hospital rating [7]. Additionally, seven questions pertain to demographics, which are used to standardize hospitals for analytic purposes and comparison [22]. The final three questions are screening items designed to direct patients to the next appropriate question.

### 2.2 Data Source

The HCAHPS data utilized in this study were sourced from two distinct public datasets available on data.cms.gov. Both datasets were collected from July 1, 2021 to June 30, 2022. Prior to public release, CMS adjusted the survey responses in these datasets to account for differences in survey administration mode and patient demographics [22].

The datasets include (1) “Hospital‐level results for the HCAHPS” and (2) “Hospital‐level results for the Prospective Payment System (PPS)‐Exempt Cancer Hospital Quality Reporting Program (PCHQR).”

The HCAHPS dataset contains responses from 4714 hospitals. The survey was given to a random sample of adult patients admitted to the medical, surgical, or maternity services within 48 h and 6 weeks following their discharge [22].

The PCHQR dataset includes responses from eleven PPS‐Exempt Cancer hospitals mandated to report all necessary quality measures to CMS under the PCHQR Program. Section 3005 of the Affordable Care Act enacted this program to serve two purposes: First, it empowers consumers by offering insights into healthcare quality, aiding them in making informed decisions. Second, it promotes awareness and encourages healthcare providers, including physicians and hospitals, to adhere to and report on best practices tailored to their specific facilities and care types [23]. For a hospital to qualify and participate in the PCHQR Program, it needs to be classified as a cancer hospital and excluded from the PPS since the beginning of its first cost reporting period on or after October 1, 1989 [24].

Both datasets included 10 HCAHPS measures consisting of 6 composite measures, 2 individual items, and 2 global items [7]. The six composites assess aspects such as the healthcare providers’ demeanor and attentiveness to patients’ concerns, the promptness of staff assistance, the effectiveness of staff communication regarding medication and essential discharge information, and patients’ understanding of posthospital care [7]. The two individual items focus on cleanliness and quietness of hospital environment. The two global items gauge patients’ overall hospital rating and their likelihood to recommend the hospital [7].

The two datasets were merged based on information about facilities, such as the facility name, address, city, state, zip code, county, HCAHPS measures, number of completed surveys, survey response rate, and HCAHPS linear mean score. The responses to the 19 substantive questions in the HCAHPS survey were scored and averaged by CMS to derive the HCAHPS linear mean scores for each HCAHPS measure [7, 22]. These scores range from 0 to 100, with a higher score indicating better patient satisfaction. Rows of the merged dataset lacking a HCAHPS linear mean score were excluded from analysis. As a result, the dataset retained the linear mean score for the following HCAHPS measures: “Cleanliness,” “Nurse communication,” “Doctor communication,” “Staff responsiveness,” “Communication about medicines,” “Discharge information,” “Care transition,” “Overall hospital rating,” “Quietness,” and “Recommend hospital.”

Rurality classification was conducted by matching the zip codes from the merged dataset with the Health Provider Shortage Area Primary Care dataset publicly accessible at data.hrsa.gov/data/download.

### 2.3 Data Analysis

RStudio was used for the statistical analysis. Descriptive statistical analyses were performed to find the number of hospitals (*n*), mean, standard deviation, median, minimum, and maximum of the linear mean scores for each HCAHPS measure. Independent sample *t*‐test was performed to compare linear mean score of each hospital indicator, stratifying by rurality. A *p*‐value of less than 0.05 was deemed significant.

## 3 Results

A total of 3248 hospitals were included in this analysis. Table 1 presents a detailed comparison of the linear mean scores for patient satisfaction across various hospital determinants, categorized by rurality. The analysis includes nine key variables: Cleanliness, Nurse communication, Doctor communication, Staff responsiveness, Communication about medicines, Discharge information, Care transition, Overall hospital rating, Quietness, and Willingness to recommend the hospital.

**TABLE 1:** Linear mean score of patient satisfaction of hospital determinants stratifying by rurality 2021–2022.

Variables	Rural hospitals (*n* = 1661)	Non‐rural hospitals (*n* = 1587)	*p*‐value
	Mean (±SD)	Mean (±SD)	
Cleanliness	86.76 (±4.16)	84.12 (±4.13)	<0.0001
Nurse communication	91.14 (±2.69)	89.32 (±2.87)	<0.0001
Doctor communication	90.79 (±2.93)	89.19 (±2.57)	<0.0001
Staff responsiveness	85.21 (±4.33)	80.75 (±4.69)	<0.0001
Communication about medicines	76.99 (±4.83)	74.41 (±4.63)	<0.0001
Discharge information	86.40 (±4.19)	84.48 (±4.06)	<0.0001
Care transition	80.34 (±3.24)	79.37 (±3.23)	<0.0001
Overall hospital rating	87.24 (±3.71)	86.11 (±4.05)	<0.0001
Quietness	82.94 (±4.61)	79.37 (±4.91)	<0.0001
Recommend hospital	85.67 (±4.78)	85.14 (±5.53)	0.0032

In the Cleanliness category, rural hospitals reported a higher mean score (*x̅* = 86.76) compared to their non‐rural counterparts (*x̅ *= 84.12) (*p* < 0.05). Similarly, Nurse communication was rated more favorably in rural settings (*x̅* = 91.14) than in non‐rural (*x̅* = 89.32) (*p *< 0.05). This pattern of rural hospitals outperforming non‐rural ones continued across other variables, including Doctor communication, Staff responsiveness, Communication about medicines, and Discharge information, with *p*‐values strongly indicating statistical significance.

The Care transition mean scores, though higher in rural hospitals (*x̅* = 80.34) compared to non‐rural (*x̅* = 79.37), displayed a relatively smaller, yet significant difference (*p* < 0.05). The Overall hospital rating followed a similar trend, with rural hospitals marginally outscoring non‐rural (*x̅* = 87.24 vs. *x̅* = 86.11), and the difference was statistically significant (*p*<0.05).

A notable disparity was observed in the Quietness parameter, where rural hospitals significantly outperformed non‐rural hospitals (*x̅* = 82.94 vs. *x̅* = 79.37) (*p* < 0.05). Lastly, the willingness to Recommend the hospital was slightly higher in rural settings (*x̅* = 85.67) compared to non‐rural (*x̅* = 85.14), with the difference being the least pronounced among all variables yet statistically significant (*p* < 0.05).

Overall, the data consistently indicate that patient satisfaction across various determinants tends to be higher in rural hospitals than non‐rural ones.

## 4 Discussion

The results demonstrated a significant difference in every hospital determinant when comparing rural and non‐rural status, with rural hospitals exhibiting a higher score across all hospital determinants. These findings challenge prior research that typically associates rural hospitals with lower levels of satisfaction [12]. For instance, a previous study using HCAHPS data from 2007 found that patients in rural hospitals were less likely to rate the cleanliness of their environment favorably, suggesting possible issues with hospital management and the availability of cleaning staff in rural healthcare facilities [11]. The higher scores in these hospital determinants in our study may indicate that there has been improvement in these aspects in more recent years. However, it has been suggested that patients in rural areas may have higher patient satisfaction than in urban areas due to lower expectations stemming from the limited availability of medical resources in rural settings [25]. If rural patients are aware of the shortage of healthcare providers, they may have greater tolerance toward subpar or poor levels of communication.

A significant trend to note is the referral of critically ill rural patients or those with complex medical conditions to larger urban hospitals rather than local rural facilities [26, 27]. This referral trend can contribute to the observed disparity in patient satisfaction scores between urban and rural hospitals in our study. This pattern is key to understanding why urban hospitals might appear to perform “worse” in our study. Research indicates that hospitals treating patients with more severely ill patients tend to receive lower scores on HCAHPS surveys [28]. These lower scores can adversely affect the reimbursements these hospitals receive from CMS, posing a considerable challenge for urban hospitals that frequently manage complex medical cases. This situation underscores the need for a more nuanced interpretation of satisfaction scores, as high scores do not always correspond with traditional measures of outcomes and safety [29]. This discrepancy highlights a notable limitation of the HCAHPS surveys: the failure to adjust for the severity of illness. Recognizing this shortcoming is crucial, highlighting the need for refinements in this evaluative framework to ensure a fair and accurate assessment of hospital performance.

Overall, the variation in patient satisfaction suggests potential inequalities in healthcare access and differences in quality of care among different populations. This emphasizes the need to examine regional inequality in healthcare delivery, as it may play a significant role in shaping disparities in overall health outcomes, including patient satisfaction. Given the clear regional disparities based on factors like rural status, a more nuanced approach is required. Instead of focusing solely on standardization of practices and policies, interventions should aim to address the underlying factors contributing to these disparities and to promote health equity. People tend to adapt to their regional conditions, so rural patients may value different attributes of healthcare delivery [30]. For example, given the long distances rural patients tend to face in accessing healthcare facilities, they may express a preference for telemedicine services. Consequently, hospitals may prioritize the maintenance of stable Wi‐Fi infrastructure over investing in physical infrastructure upgrades. Additionally, policies should not exclusively target rural versus urban areas due to multiple reasons. First, the difference between a rural area and an urban area can sometimes be vague. With various definitions of “rural” and “urban,” there is risk for patients to be incorrectly matched with a program or policy [31]. Second, not all underserved communities are found in rural locations. In fact, 29.28% of all health professional shortage areas are classified as non‐rural [32]. Therefore, addressing these disparities may require targeted resource allocation, tailored healthcare programs, and policy initiatives designed to meet the specific needs of underserved regions.

Despite the need for nuanced approaches to address the unique challenges of different healthcare settings, certain interventions can benefit both underserved areas and all hospitals. For example, hospitals will still benefit from interventions targeting effective communication with healthcare providers. There has been a growing emphasis on integrating communication training into healthcare provider curriculum, driven by studies that demonstrated improved patient satisfaction following such training compared to the control group without training [33, 34, 35]. Additionally, patients who lack a regular doctor often feel more uncomfortable asking questions and communicating with doctors [36]. Consequently, interventions should also include initiatives to establish consistent relationships with patients. It is crucial to acknowledge that clinicians in underserved areas often face higher workloads and lack of resources, factors found to be associated with burnout and job dissatisfaction [37]. Addressing better working conditions for the staff in underserved areas may positively impact the care they provide to patients, subsequently enhancing patient satisfaction.

## 5 Limitations

Given that this was a cross‐sectional study with data collection from 2021 to 2022, it limits the generalizability of this study as patient satisfaction can easily evolve within a few years due to emerging healthcare standards and technological advances that affect patient care. Additionally, the study's results are reflective solely of hospital performance within the United States, which must be considered when applying these findings to other healthcare systems or international contexts.

Additionally, the data collection period of 2021 to 2022 coincided with the ongoing COVID‐19 pandemic, presenting significant challenges for healthcare systems worldwide due to healthcare shortages and increased demands. As such, we acknowledge that our study's findings may be influenced by the unique circumstances of this period. For example, the possibility of patient transfers from rural to urban hospitals during the pandemic could further complicate the interpretation of our results. Although rural hospitals may have appeared to perform better in terms of patient satisfaction, this could be attributed to factors such as patient redistribution rather than intrinsic deficiencies in care quality. Given these considerations, we caution that our study's results may primarily reflect the conditions observed during the COVID‐19 pandemic. We anticipate that patient satisfaction levels may vary in the absence of pandemic‐related stressors, and future research is warranted to explore how these dynamics evolve over time.

Furthermore, it is important to note that although HCAHPS surveys offer valuable insights into patients’ overall perception and satisfaction levels, they inherently provide only broad implications and lack detail in identifying the specific factors contributing to low or high ratings. The survey informs us that certain aspects might be rated poorly, but they do not delve into the nuanced reason behind these ratings. This limitation hinders the ability to draw comprehensive and specific conclusions, making it challenging to formulate targeted and effective interventions based solely on HCAHPS data. Moreover, other regional factors, such as socioeconomic status and demographic composition, were not controlled in the analysis. Thus, the analysis does not fully capture the complexity of regional influences on hospital patient satisfaction. Another consideration is the exclusion of deceased patients who are unable to participate in the survey. Although CMS has attempted to adjust survey responses to account for a few differences, it is important to acknowledge that potential biases may persist. Survey response rates can vary across regions, age groups, and medical treatments.

Finally, this study reveals statistically significant differences with extremely small *p*‐values; however, the practical significance of these findings must be considered. The large sample size could have accentuated minor statistical differences that hold limited clinical or operational relevance. For example, although the “Discharge Information” scores of 86.40 and 84.48 are statistically significant, the overlap in standard deviations may imply negligible practical differences. This discrepancy underscores that statistical significance does not always equate to meaningful real‐world impacts. Clinical interpretations should therefore balance statistical outcomes with their practical implications to ensure that the findings are truly applicable.

## 6 Future Implications

The reasons provided for the results are plausible, and future research should delve deeper into why hospitals in rural settings report higher patient satisfaction ratings and whether this trend is consistent. Enhancing HCAHPS surveys with more detailed qualitative methods, such as patient interviews or focus groups, can offer a deeper insight into the factors influencing patient satisfaction and help inform more precise and impactful interventions in the future.

## 7 Conclusion

This study challenges prevailing notions by revealing higher patient satisfaction ratings in rural hospitals. Although the redirection of severely ill patients to urban settings poses challenges, it emphasizes the nuanced nature of evaluating healthcare quality. Recognizing the study limitations, future research should explore reasons behind rural hospital patients’ higher satisfaction and employ qualitative methods for targeted interventions.

## Author Contributions

**Man Hung**: Conceptualizaition; Investigation; Data curation; analysis and interpretation; writing – first draft; Editing; Review and approval. **Sharon Vu**: Data curation; analysis and interpretation; writing – first draft; Editing; Review and approval. **Logan Reese**: Data curation; analysis and interpretation; Review and approval. **Neha Patel**: Data curation; analysis and interpretation; Review and approval.

## Ethics Statement

The authors have nothing to report.

## IRB Approval

Not applicable as the study did not involve human subjects.

## Conflicts of Interest

The authors declare no conflicts of interest.

## Data Availability

Data are publicly accessible on data.cms.gov. Interested parties can access the dataset by visiting https://data.cms.gov/provider‐data/dataset/dgck‐syfz. There are no restrictions on accessing or using the data. For further inquiries or assistance with accessing the data, please refer to https://cmsqualitysupport.servicenowservices.com/qnet_qa.
